# The MHC Associated Peptide Proteomics assay is a useful tool for the non-clinical assessment of immunogenicity

**DOI:** 10.3389/fimmu.2023.1271120

**Published:** 2023-10-16

**Authors:** Wojciech Jankowski, Christopher Kidchob, Campbell Bunce, Edward Cloake, Ricardo Resende, Zuben E. Sauna

**Affiliations:** ^1^Hemostasis Branch 1, Division of Hemostasis, Office of Plasma Protein Therapeutics, Center for Biologics Evaluation and Research, Food and Drug Administration, Silver Spring, MD, United States; ^2^Abzena, Cambridge, United Kingdom

**Keywords:** therapeutic protein, immunogenicity, MAPPS, HLA, protein engineering, anti-drug antibodies

## Abstract

The propensity of therapeutic proteins to elicit an immune response, poses a significant challenge in clinical development and safety of the patients. Assessment of immunogenicity is crucial to predict potential adverse events and design safer biologics. In this study, we employed MHC Associated Peptide Proteomics (MAPPS) to comprehensively evaluate the immunogenic potential of re-engineered variants of immunogenic FVIIa analog (Vatreptacog Alfa). Our finding revealed the correlation between the protein sequence affinity for MHCII and the number of peptides identified in a MAPPS assay and this further correlates with the reduced T-cell responses. Moreover, MAPPS enable the identification of “relevant” T cell epitopes and may contribute to the development of biologics with lower immunogenic potential.

## Introduction

Therapeutic proteins are used to address serious clinical conditions and have emerged as an important class of therapeutics. Despite their many advantages over small molecule drugs, therapeutic proteins have one important drawback; protein-drugs can elicit immune responses in the patient. Anti-drug antibodies (ADAs) that do not directly affect the therapeutic protein are referred to as binding antibodies and immunogenicity risks are limited. On the other hand, neutralizing antibodies (NABs) can affect the efficacy of the medication, alter the PK/PD profile of the drug, or interact with, and neutralize endogenous proteins (1, 2). ADAs can also elicit hypersensitivity responses in the patient and be life threatening. Consequently, immunogenicity is a serious concern during the development of any therapeutic proteins and has been the subject of many white papers and guidance documents (3–7).

The development of some drugs has been discontinued due to immunogenicity issues (8–11). However, even protein drugs that are licensed and marketed continue to be sub-optimal due to immune responses in some patients (12). The evaluation of the immunogenicity risk of putative protein drugs early in drug development is thus increasingly carried out. To estimate the immunogenicity of candidate drugs several computational tools, *in vitro* and *in vivo* assays have been developed in the last two decades [for an overview see (13)].

The early (and necessary) steps in such an immune response to therapeutic proteins involve: (i) Internalization of the therapeutic protein into antigen presenting cells (APCs). (ii) Degradation of the therapeutic protein into peptide fragments. (iii) Presentation of the therapeutic peptide-derived fragments on Major Histocompatibility Complex Class II (MHCII) molecules on APCs. (iv) Recognition of the peptide-MHC-II complex by T cell receptors (TCRs) on CD4^+^ T cells. (v) Proliferation of the CD4^+^ T cells.

Most tools and assays used to assess immunogenicity risk of therapeutic proteins prior to the initiation of clinical trials interrogate one or more of the early steps in the immune response described above. For instance, computational assessments can predict with considerable accuracy the binding affinity of the therapeutic protein derived peptides to MHCII molecules which is an indicator of whether a specific peptide will be presented by the MHCII repertoire of an individual (14). Similarly, *in vitro* methods can be used to experimentally measure the peptide-MHCII affinities (15). These assessments however assume that all potential peptides can be generated from a therapeutic protein, which is not the case. Consequently, there is the possibility of overestimating immunogenicity because some peptides from the therapeutic protein are found to bind strongly to MHCII molecules, however these may never be generated by APCs (16, 17). Other methods, that determine T cell proliferation following incubation with the therapeutic protein (1, 15) involve protein internalization, peptide processing, MHCII presentation, recognition by TCRs and T cell proliferation. The drawback of these methods is that they provide information about the overall immunogenicity of the protein, however, do not allow identification of specific T cell epitopes.

Here we discuss a method that has been gaining momentum in assessing the immunogenicity of therapeutic proteins, namely, the MHC associated peptide proteomics (MAPPs) assay (18). This method is more expensive, complex and resource intense compared to other methods and it is important to demonstrate its value.

The MAPPs assay allows identification of naturally presented, therapeutic protein-derived peptides on MHC proteins. The workflow involves exposure of the full-length therapeutic protein to APCs, and the identification of peptides bound to the MHC complexes. Consequently, this is the only assay that provides information about both processing of the protein to generate peptides and the presentation of these peptides on the MHC. Nonetheless, the considerable resources demanded by a MAPPs assay necessitate that the utility of the assay is demonstrated. In this study we use well characterized variants of recombinant Factor VIIa (FVIIa) to evaluate the MAPPs assay.

Recombinant FVIIa is licensed as bypass therapy for hemophilia A patients who develop neutralizing anti-drug antibodies to Factor VIII (FVIII) (19). From an immunological perspective, patients treated with recombinant FVIIa are not deficient in FVII, they are consequently tolerized to FVII and no anti-FVIIa antidrug antibodies have been reported. However, an analog of FVIIa with 3 amino acid substitutions elicited anti-drug antibodies in 11% of the patient population during a phase 3 clinical trial (8). The clinically relevant neo-epitopes were used, *post-hoc*, to evaluate concordance between *in silico*, *in vitro* and *ex vivo* assessments and clinical immunogenicity (20). Subsequently, the immunogenic variant of FVIIa was re-engineered to be less immunogenic (21). This set of molecules; wild type FVIIa, immunogenic variant and de-immunized analogs were used to evaluate the utility of the MAPPs assay for obtaining clinically relevant information.

## Materials and methods

### Isolation of PBMCs

Peripheral blood mononuclear cells (PBMCs) were obtained from healthy community subject buffy coats (from blood drawn within 24 hours), under consent, from commercial vendors. PBMC were isolated from buffy coats using Lymphocyte separation medium (Corning, Amsterdam, The Netherlands) density centrifugation and CD8^+^ T-cell depleted using CD8^+^ RosetteSep (StemCell Technologies Inc., London, United Kingdom).

### T-cell proliferation assay

A cohort of 50 subjects was selected to best represent the number and frequency of HLA-DR and HLA-DQ allotypes expressed in the world population. PBMC were counted, viability assessed by acridine orange and propidium iodide using a Luna-FL Automated Cell Counter (Logos Biosystems, Annandale, VA), and suspended in AIM-V culture medium (Invitrogen, Paisley, United Kingdom) at 4 to 6 × 10^6^ PBMC/mL. Bulk cultures were established for each subject where cells were added to a 24-well plate (Corning Life Science) along with peptide to give a final concentration of 5 µM. For each subject a clinically relevant positive control (cells incubated with exenatide [Bydureon, AstraZeneca, United Kingdom]) and a negative control (cells incubated with culture medium alone) were also included. An additional positive control used in the assay was Keyhole limpet hemocyanin. Proliferation of CD4^+^ T cells within the culture was measured on days 5, 6, 7, and 8 poststimulation by gently resuspending the cells and removal of 3 × 100 μL samples, which were transferred to a round bottomed 96-well plate and pulsed with 0.75 μCi/well tritiated thymidine (Perkin Elmer, Buckingham, United Kingdom). After 18 hours, the cultures were harvested onto filter mats (Perkin Elmer) using a TomTec Mach III cell harvester and counts per minute (cpm) for each well determined by Meltilex (Perkin Elmer) scintillation counting on a 1450 Microbeta Wallac Trilux Liquid Scintillation Counter (Perkin Elmer) in parallax, low background counting mode. All assays were performed in triplicate. Stimulation index (SI) was calculated by dividing the average counts per minute from peptide cultures by the average counts per minute in medium control cultures.

### MAPPs assay

Monocyte derived Dendritic cells (MoDC) were prepared using PBMC from 11 donors using RoboSep™ negative human monocyte isolation kits and RoboSep™ cell isolation instrument (StemCell Technologies, Cambridge, UK) according to the manufacturer’s instructions. Monocytes were re-suspended in MoDC differentiation medium and incubated at 37°C, 5% CO2. On day 7, the test samples were added to the cells in MoDC culture medium, and incubated at 37°C, 5% CO2. Following incubation, cells were matured by the addition of LPS (Sigma Aldrich, Poole, UK) and incubated at 37°C, 5% CO2 for 18 hours. On day 8, MoDC were harvested, washed and pelleted prior to flash-freezing at -80°C and cell lysis.

Following cell lysis, the HLA-DR / peptide complexes were purified from the cell lysate by immunoprecipitation and peptides bound to HLA-DR were eluted under acidic conditions. Peptides were analysed using nano liquid chromatography coupled to an Orbitrap mass spectrometer and were identified using the Sequest algorithm, built in the Proteome Discoverer software v2.1 (ThermoFisher Scientific) against a proprietary database and the sequences of the test samples.

Once the final list of identified peptides was completed, the sequence heatmaps were generated using MATLAB (MathWorks®, Cambridge, UK) to allow visualization of the sequence location and frequency of the identified peptides.

### In silico peptide-MHC-II binding affinity computations

The set of 4 molecules; wild type FVIIa, the immunogenic variant, Vatreptacog alfa (VA), and two de-immunized analogs of VA, DI-1 and DI-2 were evaluated with their respect to common MHC-II variants. The computation involved generating overlapping 15 mer ammino acid sequences from the primary sequence of each of the 4 molecules. Using the algorithm, NetMHCIIpan 3.2, a machine learning algorithm we predicted the binding of each of the peptides generated to a set of 38 MHC-II molecules. NETMHCIIpan 3.2 utilizes training with randomly generated sets of peptides to generate binding distribution curves for each MHC-II allele (14) which permits binding affinity to be expressed as a percentile rank. Our analyses used a set of 38 DRB1 alleles which represent 99.12% of the allele coverage for the North American population. The peptide-MHC-II binding affinity data set was presented as a promiscuity score [] as a surrogate measure of immunogenicity in the population. The promiscuity score is the sum of the allele frequencies of the MHC-II alleles that binding with high affinity (percentile rank<10%).

### Density plots of peptides identified in the MAPPs assay

The probability density plot was generated individually from MAPPs data obtained for each donor. For the set of FVIIa-derived peptides identified in the MAPPs assay, the minimum percent-rank score of each peptide for that subject’s HLA alleles was estimated using netMHCIIpan version 3.2.40. The scores were plotted as histograms.

### Calculation of cluster frequencies

The frequency of each cluster in the cohort was calculated according to the equation below:


Cluster frequency=(number of donors common to a cluster÷total number of donors)×100

## Results

### Immunogenicity of proteins used in this study

**Figure 1:**
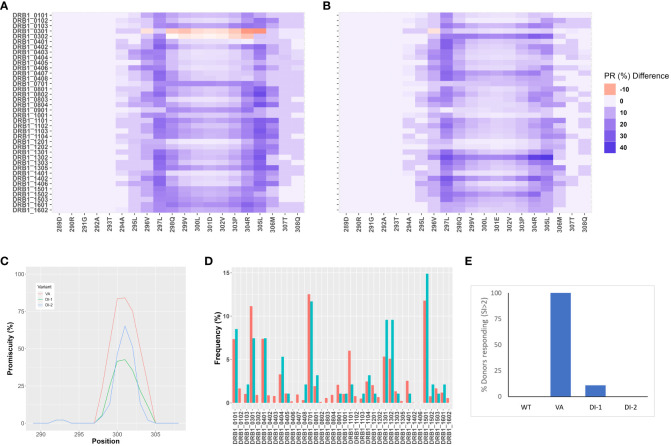
In-silico and *in vitro* immunogenicity assessments of deimmunized variants DI-1 and DI-2. **(A,B)** Peptide-MHC-II affinity for 15 mer overlapping peptides in the region of the VA mutations, E296V, and M298Q were determined as percentile ranks. The Y-axis shows the individual MHC-II DRB1 variants, and the X-axis shows the amino acid position. Each position depicts a 15 mer peptide and the amino acid depicted on the figure represents the central peptide in that peptide (i.e., position 9). The percentile rank change between VA and the two variants, DI-1 **(A)** and DI-2 **(B)** are shown. The darker blue color represents an increase in percentile rank (i.e., decrease in affinity) for the de-immunized variant as compared to VA. **(C)** Promiscuity scores (22) for VA, DI-1, and DI-2 derived 15 mer peptides in the region of the mutations introduced in VA. The promiscuity score describes the fraction of MHC-II variants a peptide binds to with high affinity (percentile score<10) weighted for the frequency with which each MHC-II variant occurs in the North American population. **(D)** Distribution of MHC-II variants in the donor cohort used for a T cell proliferation assay (results depicted in (E). The frequencies at which each MHC-II DRB1 variant occurs in the donor cohort (blue bars) and in the North American population (red bars) are shown. **(E)** Cells from the same donor cohort depicted in **(D)** were subjected to a 3H-incorporation T-cell proliferation assay. The per-cent of donors responding to each of the 4 proteins (WT, VA, DI-1, and DI-2) are depicted. Cells from a donor were considered ‘responders’ if the day 8 stimulation index (SI) value was >2 (see Methods for definition of SI).

The FVIIa analog, Vatreptacog alfa (VA) elicited anti-drug antibodies in 11% of the population in a clinical trial (8). Two deimmunized variants (DI-1 and DI-2) of VA were designed for reduced immunogenicity (21). We determined the reduction in binding affinities of DI-1 (**Figure 1A**) and DI-2 (**Figure 1B**) peptides compared to the equivalent VA peptides for common MHCII variants. Together the MHCII variants depicted in **Figures 1A, B**, occur in >90% of the North American (NA) population. Similarly, the deimmunized variants show a significant (p = 0.0113 for DI-1 and p = 0.0299 for DI-2) decrease in the promiscuity score compared to VA (**Figure 1C**). The promiscuity score describes the fraction of MHC-II variants a peptide binds to with high affinity (percentile score<10) weighted for the frequency with which each MHC-II variant occurs in the NA population (22).

We also evaluated the wild type FVIIa, the engineered analog VA and the two de-immunized variants DI-1 and DI-2 in an *in vitro* T cell proliferation assay. For the T cell proliferation assay (see Methods) we used PBMCs obtained from 50 donors. The relative frequencies of MHC-II variants in our cohort were comparable to those found in the NA population (**Figure 1D**). Cells from a donor were considered responsive in the assay if the stimulation index (SI) was >2 (see Methods for definition of SI). Consistent with clinical experience, no donors responded to the wild type FVIIa while almost all donors responded to VA. 2 and 0% of donors responded to the deimmunized variants DI-1 and DI-2, respectively (**Figure 1E**). Thus, the potential immunogenicity (based on this assay) of the deimmunized variants was comparable to that of the wild-type protein which has not been associated with immunogenicity in the clinic (23).

The four variants of FVIIa (wild type, VA, DI-1, and DI-2) have distinctive T cell mediated immune responses. The immune responses are likely based on differences in the presentation of peptides by MHC-II proteins on APCs (24). Consequently, these variants were used to assess the utility of the MAPPs assay in immunogenicity assessments.

### Mutant FVIIa peptides identified in a MAPPs assay

**Figure 2:**
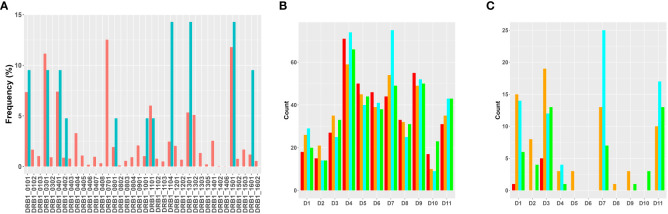
Overview of results of the MAPPs assay. **(A)** Distribution of MHC-II variants in the donor cohort used for MAPPs assays. The frequencies at which each MHC-II DRB1 variant occurs in the donor cohort (blue bars) and in the North American population (red bars) are shown. **(B)** The total number of FVIIa-derived peptides recovered from dendritic cells from each of the donors when matured in the presence of WT (red bars), VA (orange bars), DI-1 (blue bars) or DI-2 (green bars) are shown. **(C)** The total number of FVIIa-derived peptides in the region of the VA mutations (E296, and M298) recovered from dendritic cells from each of the donors when matured in the presence of WT (red bars), VA (orange bars), DI-1 (blue bars) or DI-2 (green bars) are shown.

We carried out the MAPPs assay using PBMCs from 11 donors (see Methods for details). All donors were HLA typed and the MHC-II DRB1 alleles identified in the cohort represent >75% of higher frequency alleles identified in the NA population. Moreover, the relative frequency of each MHC-II DRB1 allele in the cohort is comparable to the frequency of that allele in the NA population (**Figure 2A**). MoDC from the same cohort of donors were incubated with each of the four variants of FVIIa (wild type, VA, DI-1, and DI-2) to identify peptides presented on the MHC-II DRB1 proteins. The workflow in this study involved affinity capture of MHC-II DRB1 alleles. Thus, while the HLA typing shows the DR, DP and DQ variants for each donor, the peptides identified in the assay are only those associated with the DR alleles. We have included this limitation in the results section. Previously, we identified peptides that were presented by the DR, DP and DQ alleles separately (25). We determined that most of the peptides (~80 %) were presented by the DRB1 alleles.

The total number of sample-specific peptides identified on MHC-II proteins isolated from each donor when incubated with the four FVIIa variants are shown in **Figure 2B**. In addition to the total number of peptides, we also tabulated the number of peptides that included the E296V and M298Q mutations introduced into VA (**Figure 2C**).

**Figure 3:**
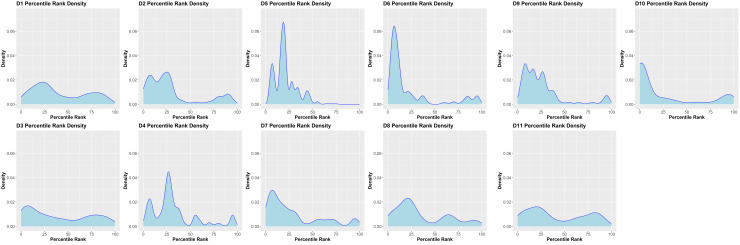
Density plots of FVIII peptides identified on donor MHC-II variants in the MAPPs assay. The distributions of percentile rank scores predicted by netMHCIIpan3.2 for all peptides found in the MAPPs assay for all 11 donors used in the study are depicted. Peptides found in the MAPPs assay were more likely to have high binding affinity to the patients’ alleles as shown by the greater probability of finding lower percentile rank scores for these peptide/MHC-II binding pairs.

We have previously demonstrated that peptides identified in a MAPPs assay have a higher affinity to the MHC-II proteins of the donor/patient (25). We estimated the peptide-MHC-II affinities for each of the peptides identified in the MAPPs assay using the MHC-II allotype of the donor. The peptide-MHC-II binding affinities were converted to a percentile rank. We demonstrate that the peptides identified in the MAPPs assay are skewed to the left of the plot (**Figure 3**), i.e., there is a greater probability of finding peptides with lower percentile rank scores (higher affinity). An overview of the characteristic of FVIIa-derived peptides identified in the MAPPs assay is provided in **Tables 1A****,** **B**.

**Table 1A:** Overview of peptides found for each donor in the MAPPS assay.

Donor	Total Number of Peptides^1^	Number of Unique Peptides^2^	Average Peptide Length^3^	Minimum Peptide Length^4^	Maximum Peptide Length^5^
**D1**	**93**	**55**	**17**	**13**	**26**
**D2**	**64**	**31**	**17**	**13**	**24**
**D3**	**120**	**74**	**17**	**13**	**25**
**D4**	**270**	**80**	**18**	**12**	**25**
**D5**	**179**	**55**	**17**	**12**	**24**
**D6**	**164**	**43**	**16**	**12**	**21**
**D7**	**222**	**96**	**17**	**10**	**25**
**D8**	**121**	**37**	**17**	**13**	**25**
**D9**	**206**	**60**	**18**	**11**	**25**
**D10**	**59**	**24**	**17**	**13**	**20**
**D11**	**152**	**72**	**16**	**11**	**26**

1. Total number of peptides found for each donor across all four variants of FVIIa.

2. Number of unique peptides found after removing duplicate sequences.

3. Average peptide length for peptides found in each donor.

4. For each donor, the peptide with the shortest length was recorded.

5. For each donor, the peptide with the longest length was recorded.

**Table 1B:** Overview of peptides found for each donor-construct pair in the MAPPS assay.

Donor Construct	Total Number of Peptides	Number of Unique Peptides	Average Peptide Length	Minimum Peptide Length	Maximum Peptide Length
**D1-DI-1**	**29**	**29**	**16**	**13**	**19**
**D1_DI-2**	**20**	**20**	**17**	**14**	**24**
**D1_VA**	**26**	**25**	**17**	**13**	**26**
**D1_WT**	**18**	**18**	**17**	**14**	**19**
**D2_DI-1**	**14**	**12**	**16**	**13**	**22**
**D2_DI-2**	**14**	**11**	**16**	**13**	**18**
**D2_VA**	**21**	**17**	**17**	**14**	**24**
**D2_WT**	**15**	**14**	**17**	**14**	**23**
**D3_DI-1**	**25**	**24**	**16**	**13**	**19**
**D3_DI-2**	**33**	**30**	**17**	**13**	**24**
**D3_VA**	**35**	**31**	**17**	**13**	**24**
**D3_WT**	**27**	**25**	**18**	**14**	**25**
**D4_DI-1**	**74**	**69**	**18**	**12**	**25**
**D4_DI-2**	**66**	**64**	**18**	**13**	**25**
**D4_VA**	**59**	**58**	**18**	**13**	**25**
**D4_WT**	**71**	**69**	**18**	**12**	**25**
**D5_DI-1**	**40**	**38**	**17**	**13**	**22**
**D5_DI-2**	**44**	**42**	**17**	**12**	**22**
**D5_VA**	**45**	**43**	**17**	**12**	**24**
**D5_WT**	**50**	**47**	**17**	**12**	**22**
**D6_DI-1**	**41**	**37**	**16**	**13**	**21**
**D6_DI-2**	**38**	**33**	**16**	**13**	**21**
**D6_VA**	**39**	**34**	**16**	**12**	**21**
**D6_WT**	**46**	**39**	**16**	**12**	**21**
**D7_DI-1**	**75**	**68**	**17**	**10**	**25**
**D7_DI-2**	**49**	**48**	**16**	**10**	**25**
**D7_VA**	**54**	**52**	**17**	**10**	**25**
**D7_WT**	**44**	**43**	**17**	**10**	**25**
**D8_DI-1**	**25**	**23**	**17**	**13**	**23**
**D8_DI-2**	**31**	**29**	**17**	**13**	**25**
**D8_VA**	**32**	**29**	**18**	**13**	**25**
**D8_WT**	**33**	**29**	**17**	**13**	**23**
**D9_DI-1**	**52**	**50**	**17**	**13**	**25**
**D9_DI-2**	**50**	**48**	**18**	**13**	**25**
**D9_VA**	**49**	**47**	**17**	**13**	**25**
**D9_WT**	**55**	**52**	**18**	**11**	**25**
**D10_DI-1**	**9**	**6**	**18**	**16**	**20**
**D10_DI-2**	**23**	**21**	**17**	**13**	**20**
**D10_VA**	**10**	**7**	**18**	**14**	**20**
**D10_WT**	**17**	**16**	**16**	**14**	**20**
**D11_DI-1**	**43**	**40**	**16**	**11**	**20**
**D11_DI-2**	**43**	**40**	**16**	**13**	**26**
**D11_VA**	**35**	**33**	**17**	**13**	**26**
**D11_WT**	**31**	**29**	**17**	**13**	**25**

1. Total number of peptides found for each donor and FVIIa protein combination.

2. Number of unique peptides found after removing duplicate sequences.

3. Average peptide length for peptides found in each donor and FVIII protein.

4. For each donor and FVIII protein, the peptide with the shortest length was recorded.

5. For each donor and FVIII protein, the peptide with the longest length was recorded.

### FVIIa peptides identified in the MAPPs assay locate to 10 clusters

**Figure 4:**
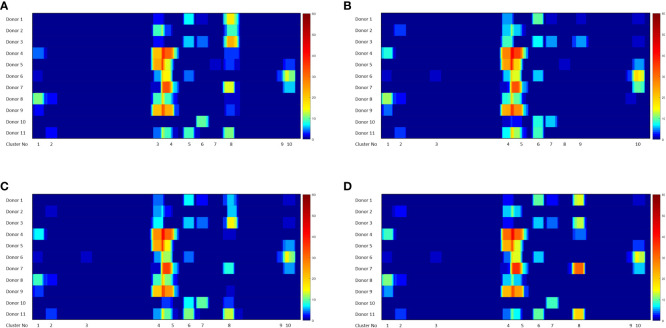
Heatmaps showing FVIIa-derived peptides identified in the MAPPs assay. The position of the FVIIa amino acid sequence and all identified clusters are shown on the X-axis. The heatmaps depicts the number of peptides identified in the MAPPs assay at each position following incubation with VA **(A)**, WT **(B)**, DI-1 **(C)** and DI-2 **(D)** proteins. Note that 8 clusters of peptides (shown on the figure) were identified for all treatments.

The heat maps depicted in **Figure 4** show all the FVIIa-derived peptides identified in the MAPPs assay following incubation of APCs with the FVIIa variants. All peptides were incubated with cells from the same cohort of donors, i.e., an identical distribution of HLA alleles. Although the relative number of peptides varies, all FVIIa variants result in the identification of peptides from the same 10 clusters. This suggests similar processing of the FVIIa variants by the proteolytic machinery of the APCs. This cohort of donors all have a functional FVIIa. Thus, the peptides are mostly self, i.e., they have the same sequence as the endogenous FVIIa expressed by the donor. The peptides found in cluster 8, include mutations in the wild-type sequence to generate the VA and DI-1 and DI-2 variants. These foreign/non-self-peptides are the ones that are relevant vis-à-vis immunogenicity as these are most likely to initiate an immune response to the FVIIa variants. The peptides identified in cluster 8 are listed in **Tables 2A****–****D**. It is important to note that, compared to VA, far fewer wild-type peptides are presented by the APCs. This finding demonstrates that the mutations introduced in the VA variant enhance the processing and/or presentation of FVIIa-peptides by the APCs. That the VA peptides are also foreign peptides further increases the probability of these peptides being identified by T cell receptors (TCRs) resulting in a potential initiation of the immune response.

**Table 2A:** Peptides from the Wild Type FVII found in the MAPPS assay and associated HLA alleles.

WT	Halotypes						
Sequences	DRB1	DRB1	DRB3	DRB3	DRB4	DQB1	DQB1
**DRGATALELMVLNVPRLMTQD**	**03:01:01:01**	**11:04:01**	**01:01:02:01**	**02:02:01:01**	**N/A**	**2:01:01**	**06:02:01:01**
**DRGATALELMVLNVPRLMTQDCLQ**	**03:01:01:01**	**11:04:01**	**01:01:02:01**	**02:02:01:01**	**N/A**	**2:01:01**	**06:02:01:01**
**DRGATALELMVLNVPRLMTQDCLQQ**	**03:01:01:01**	**11:04:01**	**01:01:02:01**	**02:02:01:01**	**N/A**	**2:01:01**	**06:02:01:01**
**LELMVLNVPRLMTQD**	**04:01:01:01** **03:01:01:01**	**11:04:01** **N/A**	**02:02:01:01** **01:01:02:01**	**N/A** **N/A**	**01:03:01:01** **N/A**	**03:01:01:01** **02:01:01**	**N/A** **06:02:01:01**
**LELMVLNVPRLMTQDC**	**03:01:01:01**	**11:04:01**	**01:01:02:01**	**02:02:01:01**	**N/A**	**2:01:01**	**06:02:01:01**

N/A, Not Available.

**Table 2B:** Peptides from VA construct found in the MAPPS assay and associated HLA alleles.

VA	Halotypes							
Sequence	DRB1	DRB1	DRB3	DRB3	DRB4	DRB5	DQB1	DQB1
**LLDRGATALVLQVLNVPRL**	**4:02:01**	**13:01:01:01**	**01:01:02:01**	**N/A**	**01:03:01:01**	**N/A**	**03:02:01:01**	**06:03:01:01**
**LDRGATALVLQVLNVPR**	**04:01:01:01**	**15:01:01:01**	**N/A**	**N/A**	**01:03:01:01**	**1:01:01**	**03:02:01:01**	**06:02:01:01**
**LDRGATALVLQVLNVPRL**	**04:01:01:01**	**15:01:01:01**	**N/A**	**N/A**	**01:03:01:01**	**1:01:01**	**03:02:01:01**	**06:02:01:01**
**LDRGATALVLQVLNVPRLM**	**4:02:01**	**13:01:01:01**	**01:01:02:01**	**N/A**	**01:03:01:01**	**N/A**	**03:02:01:01**	**06:03:01:01**
**DRGATALVLQVLNVPR**	**04:01:01:01** **03:01:01:01** **04:02:01**	**11:04:01** **N/A** **13:01:01:01**	**02:02:01:01** **01:01:02:01**	**N/A** **N/A** **N/A**	**01:03:01:01** **N/A** **N/A**	**N/A** **N/A** **N/A**	**03:01:01:01** **02:01:01** **03:02:01:01**	**N/A** **06:02:01:01** **06:03:01:01**
**DRGATALVLQVLNVPRL**	**04:01:01:01** **03:01:01:01** **04:02:01** **15:01:01:01**	**11:04:01** **N/A** **13:01:01:01** **N/A**	**02:02:01:01** **01:01:02:01** **N/A** **N/A**	**N/A** **N/A** **N/A** **N/A**	**01:03:01:01** **N/A** **N/A** **N/A**	**N/A** **N/A** **N/A** **01:01:01**	**03:01:01:01** **02:01:01** **03:02:01:01** **N/A**	**N/A** **06:02:01:01** **06:03:01:01** **N/A**
**DRGATALVLQVLNVPRLM**	**04:01:01:01** **03:01:01:01** **01:01:01** **04:02:01** **15:01:01:01**	**11:04:01** **N/A** **10:01:01:01** **13:01:01:01** **N/A**	**02:02:01:01** **01:01:02:01** **N/A** **N/A** **N/A**	**N/A** **N/A** **N/A** **N/A** **N/A**	**01:03:01:01** **N/A** **N/A** **N/A** **N/A**	**N/A** **N/A** **N/A** **N/A** **01:01:01**	**03:01:01:01** **02:01:01** **05:01:01:01** **03:02:01:01** **N/A**	**N/A** **06:02:01:01** **N/A** **06:03:01:01** **N/A**
**DRGATALVLQVLNVPRLMT**	**4:02:01**	**13:01:01:01**	**01:01:02:01**	**N/A**	**01:03:01:01**	**N/A**	**03:02:01:01**	**06:03:01:01**
**DRGATALVLQVLNVPRLMTQDCLQ**	**11:04:01** **03:01:01:01**	**15:01:01:01** **N/A**	**02:02:01:01** **01:01:02:01**	**N/A** **N/A**	**N/A** **N/A**	**01:01:01** **N/A**	**03:01:01:01** **02:01:01**	**06:02:01:01** **N/A**
**DRGATALVLQVLNVPRLMTQDCLQQ**	**04:01:01:01**	**11:04:01**	**02:02:01:01**	**N/A**	**01:03:01:01**	**N/A**	**03:01:01:01**	**N/A**
**DRGATALVLQVLNVPRLMTQDCLQQS**	**04:01:01:01**	**11:04:01**	**02:02:01:01**	**N/A**	**01:03:01:01**	**N/A**	**03:01:01:01**	**N/A**
**RGATALVLQVLNVPRL**	**04:02:01** **04:01:01:01**	**13:01:01:01** **15:01:01:01**	**01:01:02:01** **N/A**	**N/A** **N/A**	**01:03:01:01** **N/A**	**N/A** **01:01:01**	**03:02:01:01** **06:02:01:01**	**06:03:01:01** **N/A**
**RGATALVLQVLNVPRLM**	**4:02:01**	**13:01:01:01**	**01:01:02:01**	**N/A**	**01:03:01:01**	**N/A**	**03:02:01:01**	**06:03:01:01**
**GATALVLQVLNVPR**	**04:01:01:01** **03:01:01:01** **04:02:01** **15:01:01:01**	**11:04:01** **N/A** **13:01:01:01** **N/A**	**02:02:01:01** **01:01:02:01** **N/A** **N/A**	**N/A** **N/A** **N/A** **N/A**	**01:03:01:01** **N/A** **N/A** **N/A**	**N/A** **N/A** **N/A** **01:01:01**	**03:01:01:01** **02:01:01** **03:02:01:01** **N/A**	**N/A** **06:02:01:01** **06:03:01:01** **N/A**
**GATALVLQVLNVPRL**	**04:01:01:01** **03:01:01:01** **04:02:01** **15:01:01:01**	**11:04:01** **N/A** **13:01:01:01** **N/A**	**02:02:01:01** **01:01:02:01** **N/A** **N/A**	**N/A** **N/A** **N/A** **N/A**	**01:03:01:01** **N/A** **N/A** **N/A**	**N/A** **N/A** **N/A** **01:01:01**	**03:01:01:01** **02:01:01** **03:02:01:01** **N/A**	**N/A** **06:02:01:01** **06:03:01:01** **N/A**
**GATALVLQVLNVPRLM**	**04:01:01:01**	**15:01:01:01**	**N/A**	**N/A**	**01:03:01:01**	**1:01:01**	**03:02:01:01**	**06:02:01:01**
**GATALVLQVLNVPRLMTQDCLQ**	**04:01:01:01** **15:01:01:01** **03:01:01:01** **13:01:01:01**	**11:04:01** **N/A** **N/A** **16:01:01**	**02:02:01:01** **N/A** **01:01:02:01** **N/A**	**N/A** **N/A** **N/A** **N/A**	**01:03:01:01** **N/A** **N/A** **02:02:01**	**N/A** **01:01:01** **N/A** **N/A**	**03:01:01:01** **06:02:01:01** **02:01:01** **05:02:01:01**	**N/A** **N/A** **N/A** **06:03:01:01**
**GATALVLQVLNVPRLMTQDCLQQ**	**13:01:01:01**	**16:01:01**	**01:01:02:01**	**N/A**	**N/A**	**2:02:01**	**05:02:01:01**	**06:03:01:01**
**GATALVLQVLNVPRLMTQDCLQQS**	**04:01:01:01** **15:01:01:01** **03:01:01:01** **13:01:01:01** **01:01:01** **04:02:01** **11:01:01:01**	**11:04:01** **N/A** **N/A** **16:01:01** **10:01:01:01** **N/A** **N/A**	**02:02:01:01** **N/A** **01:01:02:01** **N/A** **N/A** **N/A** **N/A**	**N/A** **N/A** **N/A** **N/A** **N/A** **N/A** **N/A**	**01:03:01:01** **N/A** **N/A** **N/A** **N/A** **N/A** **N/A**	**N/A** **01:01:01** **N/A** **02:02:01** **N/A** **N/A** **N/A**	**03:01:01:01** **06:02:01:01** **02:01:01** **05:02:01:01** **05:01:01:01** **03:02:01:01** **06:01:01:01**	**N/A** **N/A** **N/A** **06:03:01:01** **N/A** **N/A**
**ATALVLQVLNVPR**	**04:02:01** **04:01:01:01**	**13:01:01:01** **15:01:01:01**	**01:01:02:01** **N/A**	**N/A** **N/A**	**01:03:01:01** **N/A**	**N/A** **N/A**	**03:02:01:01** **01:01:01**	**06:03:01:01** **06:02:01:01**
**ATALVLQVLNVPRL**	**04:02:01** **04:01:01:01**	**13:01:01:01** **15:01:01:01**	**01:01:02:01** **N/A**	**N/A** **N/A**	**01:03:01:01** **N/A**	**N/A** **01:01:01**	**03:02:01:01** **06:02:01:01**	**06:03:01:01** **N/A**
**ATALVLQVLNVPRLMTQDCLQQS**	**11:04:01**	**15:01:01:01**	**02:02:01:01**	**N/A**	**N/A**	**1:01:01**	**03:01:01:01**	**06:02:01:01**
**TALVLQVLNVPRL**	**1:01:01**	**10:01:01:01**	**N/A**	**N/A**	**N/A**	**N/A**	**05:01:01:01**	**N/A**
**TALVLQVLNVPRLM**	**1:01:01**	**16:01:01**	**N/A**	**N/A**	**N/A**	**2:02:01**	**05:01:01:01**	**05:02:01:01**
**ALVLQVLNVPRLM**	**1:01:01**	**16:01:01**	**N/A**	**N/A**	**N/A**	**2:02:01**	**05:01:01:01**	**05:02:01:01**
**ALVLQVLNVPRLMTQD**	**03:01:01:01**	**11:04:01**	**01:01:02:01**	**02:02:01:01**	**N/A**	**N/A**	**2:01:01**	**06:02:01:01**
**ALVLQVLNVPRLMTQDCL**	**04:01:01:01**	**11:04:01**	**02:02:01:01**	**N/A**	**01:03:01:01**	**N/A**	**03:01:01:01**	**N/A**
**ALVLQVLNVPRLMTQDCLQ**	**04:01:01:01** **03:01:01:01**	**11:04:01** **N/A**	**02:02:01:01** **01:01:02:01**	**N/A** **N/A**	**01:03:01:01** **N/A**	**N/A** **N/A**	**03:01:01:01** **02:01:01**	**N/A** **06:02:01:01**
**ALVLQVLNVPRLMTQDCLQQS**	**03:01:01:01**	**11:04:01**	**01:01:02:01**	**02:02:01:01**	**N/A**	**N/A**	**2:01:01**	**06:02:01:01**
**LVLQVLNVPRLMTQ**	**03:01:01:01**	**11:04:01**	**01:01:02:01**	**02:02:01:01**	**N/A**	**N/A**	**2:01:01**	**06:02:01:01**
**LVLQVLNVPRLMTQD**	**04:01:01:01** **15:01:01:01** **03:01:01:01**	**11:04:01** **N/A** **N/A**	**02:02:01:01** **N/A** **01:01:02:01**	**N/A** **N/A** **N/A**	**01:03:01:01** **N/A** **N/A**	**N/A** **01:01:01** **N/A**	**03:01:01:01** **06:02:01:01** **02:01:01**	**N/A** **N/A** **N/A**
**LVLQVLNVPRLMTQDC**	**11:04:01**	**15:01:01:01**	**02:02:01:01**	**N/A**	**N/A**	**1:01:01**	**03:01:01:01**	**06:02:01:01**
**LVLQVLNVPRLMTQDCL**	**04:01:01:01** **03:01:01:01**	**11:04:01** **N/A**	**02:02:01:01** **01:01:02:01**	**N/A** **N/A**	**01:03:01:01** **N/A**	**N/A** **N/A**	**03:01:01:01** **02:01:01**	**N/A** **06:02:01:01**
**LVLQVLNVPRLMTQDCLQ**	**11:04:01** **03:01:01:01**	**15:01:01:01** **N/A**	**02:02:01:01** **01:01:02:01**	**N/A** **N/A**	**N/A** **N/A**	**01:01:01** **N/A**	**03:01:01:01** **02:01:01**	**06:02:01:01** **N/A**
**VLQVLNVPRLMTQ**	**04:01:01:01** **03:01:01:01**	**11:04:01** **N/A**	**02:02:01:01** **01:01:02:01**	**N/A** **N/A**	**01:03:01:01** **N/A**	**N/A** **N/A**	**03:01:01:01** **02:01:01**	**N/A** **06:02:01:01**
**VLQVLNVPRLMTQD**	**04:01:01:01** **15:01:01:01** **03:01:01:01**	**11:04:01** **N/A** **N/A**	**02:02:01:01** **N/A** **01:01:02:01**	**N/A** **N/A** **N/A**	**01:03:01:01** **N/A** **N/A**	**N/A** **01:01:01** **N/A**	**03:01:01:01** **06:02:01:01** **02:01:01**	**N/A** **N/A** **N/A**
**VLQVLNVPRLMTQDC**	**03:01:01:01**	**11:04:01**	**01:01:02:01**	**02:02:01:01**	**N/A**	**N/A**	**2:01:01**	**06:02:01:01**
**VLQVLNVPRLMTQDCL**	**03:01:01:01**	**11:04:01**	**01:01:02:01**	**02:02:01:01**	**N/A**	**N/A**	**2:01:01**	**06:02:01:01**

N/A, Not Available.

**Table 2C:** Peptides from the DI-1 construct found in the MAPPS assay and associated HLA alleles.

DI-1	Halotypes								
Sequence	DRB1	DRB1	DRB3	DRB3	DRB4	DRB5	DRB5	DQB1	DQB1
**VSGWGQLLDRGATALVLQVLDVPR**	**4:02:01**	**13:01:01:01**	**01:01:02:01**	**N/A**	**01:03:01:01**	**N/A**	**N/A**	**03:02:01:01**	**06:03:01:01**
**GWGQLLDRGATALVLQVLDVPR**	**4:02:01**	**13:01:01:01**	**01:01:02:01**	**N/A**	**01:03:01:01**	**N/A**	**N/A**	**03:02:01:01**	**06:03:01:01**
**LLDRGATALVLQVLDVPR**	**04:01:01:01**	**15:01:01:01**	**N/A**	**N/A**	**01:03:01:01**	**N/A**	**1:01:01**	**03:02:01:01**	**06:02:01:01**
**LLDRGATALVLQVLDVPRL**	**4:02:01**	**13:01:01:01**	**01:01:02:01**	**N/A**	**01:03:01:01**	**N/A**	**N/A**	**03:02:01:01**	**06:03:01:01**
**LLDRGATALVLQVLDVPRLM**	**4:02:01**	**13:01:01:01**	**01:01:02:01**	**N/A**	**01:03:01:01**	**N/A**	**N/A**	**03:02:01:01**	**06:03:01:01**
**LDRGATALVLQVLDVPR**	**04:01:01:01** **04:02:01** **15:01:01:01**	**11:04:01** **N/A** **N/A**	**02:02:01:01** **13:01:01:01** **N/A**	**N/A** **01:01:02:01** **N/A**	**01:03:01:01** **N/A** **N/A**	**N/A** **N/A** **01:01:01**	**N/A** **N/A** **N/A**	**03:01:01:01** **03:01:01:01** **06:02:01:01**	**N/A** **06:03:01:01** **N/A**
**LDRGATALVLQVLDVPRL**	**04:01:01:01** **03:01:01:01** **04:02:01** **15:01:01:01**	**11:04:01** **N/A** **13:01:01:01** **N/A**	**02:02:01:01** **01:01:02:01** **N/A** **N/A**	**N/A** **N/A** **N/A** **N/A**	**01:03:01:01** **N/A** **N/A** **N/A**	**N/A** **N/A** **N/A** **01:01:01**	**N/A** **N/A** **N/A** **N/A**	**03:01:01:01** **02:01:01** **03:02:01:01** **N/A**	**N/A** **06:02:01:01** **06:03:01:01** **N/A**
**LDRGATALVLQVLDVPRLM**	**03:01:01:01** **04:02:01** **04:01:01:01**	**11:04:01** **13:01:01:01** **15:01:01:01**	**01:01:02:01** **N/A** **N/A**	**02:02:01:01** **N/A** **N/A**	**N/A** **01:03:01:01** **N/A**	**N/A** **N/A** **01:01:01**	**N/A** **N/A** **N/A**	**02:01:01** **03:02:01:01** **N/A**	**06:02:01:01** **06:03:01:01** **N/A**
**DRGATALVLQVLDVPR**	**04:01:01:01** **03:01:01:01** **04:02:01** **15:01:01:01**	**11:04:01** **N/A** **13:01:01:01** **N/A**	**02:02:01:01** **01:01:02:01** **N/A** **N/A**	**N/A** **N/A** **N/A** **N/A**	**01:03:01:01** **N/A** **N/A** **N/A**	**N/A** **N/A** **N/A** **01:01:01**	**N/A** **N/A** **N/A** **N/A**	**03:01:01:01** **02:01:01** **03:02:01:01** **N/A**	**N/A** **06:02:01:01** **06:03:01:01** **N/A**
**DRGATALVLQVLDVPRL**	**04:01:01:01** **03:01:01:01** **04:02:01**	**11:04:01** **N/A** **13:01:01:01**	**02:02:01:01** **01:01:02:01** **N/A**	**N/A** **N/A** **N/A**	**01:03:01:01** **N/A** **N/A**	**N/A** **N/A** **N/A**	**N/A** **N/A** **N/A**	**03:01:01:01** **02:01:01** **03:02:01:01**	**N/A** **06:02:01:01** **06:03:01:01**
**DRGATALVLQVLDVPRLM**	**04:01:01:01** **03:01:01:01** **04:02:01** **15:01:01:01**	**11:04:01** **N/A** **13:01:01:01** **N/A**	**02:02:01:01** **01:01:02:01** **N/A** **N/A**	**N/A** **N/A** **N/A** **N/A**	**01:03:01:01** **N/A** **N/A** **N/A**	**N/A** **N/A** **N/A** **01:01:01**	**N/A** **N/A** **N/A** **N/A**	**03:01:01:01** **02:01:01** **03:02:01:01** **N/A**	**N/A** **06:02:01:01** **06:03:01:01** **N/A**
**DRGATALVLQVLDVPRLMT**	**04:01:01:01** **03:01:01:01** **04:02:01** **15:01:01:01**	**11:04:01** **N/A** **13:01:01:01** **N/A**	**02:02:01:01** **01:01:02:01** **N/A** **N/A**	**N/A** **N/A** **N/A** **N/A**	**01:03:01:01** **N/A** **N/A** **N/A**	**N/A** **N/A** **N/A** **01:01:01**	**N/A** **N/A** **N/A** **N/A**	**03:01:01:01** **02:01:01** **03:02:01:01** **N/A**	**N/A** **06:02:01:01** **06:03:01:01** **N/A**
**DRGATALVLQVLDVPRLMTQ**	**13:01:01:01** **04:02:01** **04:01:01:01**	**16:01:01** **N/A** **15:01:01:01**	**01:01:02:01** **N/A** **N/A**	**N/A** **N/A** **N/A**	**N/A** **01:03:01:01** **N/A**	**02:02:01** **N/A** **01:01:01**	**N/A** **N/A** **N/A**	**05:02:01:01** **03:02:01:01** **06:02:01:01**	**06:03:01:01** **N/A** **N/A**
**DRGATALVLQVLDVPRLMTQD**	**13:01:01:01**	**16:01:01**	**01:01:02:01**	**N/A**	**N/A**	**2:02:01**	**N/A**	**05:02:01:01**	**06:03:01:01**
**DRGATALVLQVLDVPRLMTQDC**	**13:01:01:01**	**16:01:01**	**01:01:02:01**	**N/A**	**N/A**	**2:02:01**	**N/A**	**05:02:01:01**	**06:03:01:01**
**RGATALVLQVLDVPR**	**04:01:01:01** **04:02:01** **15:01:01:01**	**11:04:01** **13:01:01:01** **N/A**	**02:02:01:01** **01:01:02:01** **N/A**	**N/A** **N/A** **N/A**	**01:03:01:01** **N/A** **N/A**	**N/A** **N/A** **01:01:01**	**N/A** **N/A** **N/A**	**03:01:01:01** **03:02:01:0106:02:01:01**	**N/A** **06:03:01:01** **N/A**
**RGATALVLQVLDVPRL**	**4:02:01**	**13:01:01:01**	**01:01:02:01**	**N/A**	**01:03:01:01**	**N/A**	**N/A**	**03:02:01:01**	**06:03:01:01**
**RGATALVLQVLDVPRLM**	**04:01:01:01** **04:02:01** **15:01:01:01**	**11:04:01** **13:01:01:01** **N/A**	**02:02:01:01** **01:01:02:01** **N/A**	**N/A** **N/A** **N/A**	**01:03:01:01** **N/A** **N/A**	**N/A** **N/A** **01:01:01**	**N/A** **N/A** **N/A**	**03:01:01:01** **03:02:01:01** **06:02:01:01**	**N/A** **06:03:01:01** **N/A**
**GATALVLQVLDVP**	**4:02:01**	**13:01:01:01**	**01:01:02:01**	**N/A**	**01:03:01:01**	**N/A**	**N/A**	**03:02:01:01**	**06:03:01:01**
**GATALVLQVLDVPR**	**04:01:01:01** **03:01:01:01** **04:02:01** **15:01:01:01**	**11:04:01** **N/A** **13:01:01:01** **N/A**	**02:02:01:01** **01:01:02:01** **N/A** **N/A**	**N/A** **N/A** **N/A** **N/A**	**01:03:01:01** **N/A** **N/A** **N/A**	**N/A** **N/A** **N/A** **01:01:01**	**N/A** **N/A** **N/A** **N/A**	**03:01:01:01** **02:01:01** **03:02:01:01** **N/A**	**N/A** **06:02:01:01** **06:03:01:01** **N/A**
**GATALVLQVLDVPRL**	**04:01:01:01** **03:01:01:01** **04:02:01** **15:01:01:01**	**11:04:01** **N/A** **13:01:01:01** **N/A**	**02:02:01:01** **01:01:02:01** **N/A** **N/A**	**N/A** **N/A** **N/A** **N/A**	**01:03:01:01** **N/A** **N/A** **N/A**	**N/A** **N/A** **N/A** **01:01:01**	**N/A** **N/A** **N/A** **N/A**	**03:01:01:01** **02:01:01** **03:02:01:01** **N/A**	**N/A** **06:02:01:01** **06:03:01:01** **N/A**
**GATALVLQVLDVPRLM**	**04:01:01:01** **04:02:01** **15:01:01:01** **N/A**	**11:04:01** **13:01:01:01** **N/A** **N/A**	**02:02:01:01** **01:01:02:01** **N/A** **N/A**	**N/A** **N/A** **N/A** **N/A**	**01:03:01:01** **N/A** **N/A** **N/A**	**N/A** **N/A** **01:01:01** **N/A**	**N/A** **N/A** **N/A** **N/A**	**03:01:01:01** **03:02:01:01** **06:02:01:01** **N/A**	**N/A** **06:03:01:01** **N/A** **N/A**
**ATALVLQVLDVPR**	**04:01:01:01** **03:01:01:01** **04:02:01** **15:01:01:01**	**11:04:01** **N/A** **13:01:01:01** **N/A**	**02:02:01:01** **01:01:02:01** **N/A** **N/A**	**N/A** **N/A** **N/A** **N/A**	**01:03:01:01** **N/A** **N/A** **N/A**	**N/A** **N/A** **N/A** **01:01:01**	**N/A** **N/A** **N/A** **N/A**	**03:01:01:01** **02:01:01** **03:02:01:01** **N/A**	**N/A** **06:02:01:01** **06:03:01:01** **N/A**
**ATALVLQVLDVPRL**	**04:01:01:01** **03:01:01:0104:02:01** **15:01:01:01**	**11:04:01** **N/A** **13:01:01:01** **N/A**	**02:02:01:01** **01:01:02:01** **N/A** **N/A**	**N/A** **N/A** **N/A** **N/A**	**01:03:01:01** **N/A** **N/A** **N/A**	**N/A** **N/A** **N/A** **01:01:01**	**N/A** **N/A** **N/A** **N/A**	**03:01:01:01** **02:01:01** **03:02:01:01** **N/A**	**N/A** **06:02:01:0106:03:01:01** **N/A**
**ATALVLQVLDVPRLM**	**04:02:01** **N/A** **N/A** **N/A**	**13:01:01:01** **N/A** **N/A** **N/A**	**01:01:02:01** **N/A** **N/A** **N/A**	**N/A** **N/A** **N/A** **N/A**	**01:03:01:01** **N/A** **N/A** **N/A**	**N/A** **N/A** **N/A** **N/A**	**N/A** **N/A** **N/A** **N/A**	**03:02:01:01** **N/A** **N/A** **N/A**	**06:03:01:01** **N/A** **N/A** **N/A**
**TALVLQVLDVPR**	**04:02:01** **N/A** **N/A** **N/A**	**13:01:01:01** **N/A** **N/A** **N/A**	**01:01:02:01** **N/A** **N/A** **N/A**	**N/A** **N/A** **N/A** **N/A**	**01:03:01:01** **N/A** **N/A** **N/A**	**N/A** **N/A** **N/A** **N/A**	**N/A** **N/A** **N/A** **N/A**	**03:02:01:01** **N/A** **N/A** **N/A**	**06:03:01:01** **N/A** **N/A** **N/A**
**TALVLQVLDVPRL**	**04:02:01** **04:01:01:01** **N/A** **N/A**	**13:01:01:01** **15:01:01:01** **N/A** **N/A**	**01:01:02:01** **N/A** **N/A** **N/A**	**N/A** **N/A** **N/A** **N/A**	**01:03:01:01** **N/A** **N/A** **N/A**	**N/A** **01:01:01** **N/A** **N/A**	**N/A** **N/A** **N/A** **N/A**	**03:02:01:01** **06:02:01:01** **N/A** **N/A**	**06:03:01:01** **N/A** **N/A** **N/A**
**TALVLQVLDVPRLMTQ**	**13:01:01:01** **N/A** **N/A** **N/A**	**16:01:01** **N/A** **N/A** **N/A**	**01:01:02:01** **N/A** **N/A** **N/A**	**N/A** **N/A** **N/A** **N/A**	**N/A** **N/A** **N/A** **N/A**	**02:02:01** **N/A** **N/A** **N/A**	**N/A** **N/A** **N/A** **N/A**	**05:02:01:01** **N/A** **N/A** **N/A**	**06:03:01:01** **N/A** **N/A** **N/A**
**ALVLQVLDVPR**	**04:02:01** **04:01:01:01** **N/A** **N/A**	**13:01:01:01** **15:01:01:01** **N/A** **N/A**	**01:01:02:01** **N/A** **N/A** **N/A**	**N/A** **N/A** **N/A** **N/A**	**01:03:01:01** **N/A** **N/A** **N/A**	**N/A** **01:01:01** **N/A**	**N/A** **N/A** **N/A** **N/A**	**03:02:01:01** **06:02:01:01** **N/A** **N/A**	**06:03:01:01** **N/A** **N/A** **N/A**
**LVLQVLDVPRLMTQD**	**03:01:01:01** **N/A** **N/A** **N/A**	**11:04:01** **N/A** **N/A** **N/A**	**01:01:02:01** **N/A** **N/A** **N/A**	**02:02:01:01** **N/A** **N/A**	**N/A** **N/A** **N/A** **N/A**	**N/A** **N/A** **N/A** **N/A**	**N/A** **N/A** **N/A** **N/A**	**02:01:01** **N/A** **N/A** **N/A**	**06:02:01:01** **N/A** **N/A** **N/A**
**VLQVLDVPRLMTQD**	**04:01:01:01** **03:01:01:01** **N/A** **N/A**	**11:04:01** **N/A** **N/A** **N/A**	**02:02:01:01** **01:01:02:01** **N/A** **N/A**	**N/A** **N/A** **N/A** **N/A**	**01:03:01:01** **N/A** **N/A** **N/A**	**N/A** **N/A** **N/A** **N/A**	**N/A** **N/A** **N/A** **N/A**	**03:01:01:01** **02:01:01** **N/A** **N/A**	**N/A** **06:02:01:01** **N/A** **N/A**

N/A, Not Available.

**Table 2D:** Peptides from the DI-2 construct found in the MAPPS assay and associated HLA alleles.

DI-2	Halotypes							
Sequence	DRB1	DRB1	DRB3	DRB3	DRB4	DRB5	DQB1	DQB1
**LDRGATALVLQVLEVPR**	**04:01:01:01** **N/A** **N/A** **N/A**	**15:01:01:01** **N/A** **N/A** **N/A**	**N/A** **N/A** **N/A** **N/A**	**N/A** **N/A** **N/A** **N/A**	**01:03:01:01** **N/A** **N/A** **N/A**	**01:01:01** **N/A** **N/A** **N/A**	**03:02:01:01** **N/A** **N/A** **N/A**	**06:02:01:01** **N/A** **N/A** **N/A**
**LDRGATALVLQVLEVPRL**	**04:02:01** **04:01:01:01** **N/A** **N/A**	**13:01:01:01** **15:01:01:01** **N/A** **N/A**	**01:01:02:01** **N/A** **N/A** **N/A**	**N/A** **N/A** **N/A** **N/A**	**01:03:01:01** **N/A** **N/A** **N/A**	**N/A** **01:01:01** **N/A** **N/A**	**03:02:01:01** **06:02:01:01** **N/A** **N/A**	**06:03:01:01** **N/A** **N/A** **N/A**
**DRGATALVLQVLE**	**04:01:01:01** **N/A** **N/A** **N/A**	**15:01:01:01** **N/A** **N/A** **N/A**	**N/A** **N/A** **N/A** **N/A**	**N/A** **N/A** **N/A** **N/A**	**01:03:01:01** **N/A** **N/A** **N/A**	**01:01:01** **N/A** **N/A** **N/A**	**03:02:01:01** **N/A** **N/A** **N/A**	**06:02:01:01** **N/A** **N/A** **N/A**
**DRGATALVLQVLEVPR**	**04:01:01:01** **03:01:01:01** **04:02:01** **15:01:01:01**	**11:04:01** **N/A** **13:01:01:01** **N/A**	**02:02:01:01** **01:01:02:01** **N/A** **N/A**	**N/A** **N/A** **N/A** **N/A**	**01:03:01:01** **N/A** **N/A** **N/A**	**N/A** **N/A** **N/A** **01:01:01**	**03:01:01:01** **02:01:01** **03:02:01:01** **N/A**	**N/A** **06:02:01:01** **06:03:01:01** **N/A**
**DRGATALVLQVLEVPRL**	**04:01:01:01** **N/A** **04:02:01** **15:01:01:01**	**11:04:01** **N/A** **13:01:01:01** **N/A**	**02:02:01:01** **01:01:02:01** **N/A** **N/A**	**N/A** **N/A** **N/A** **N/A**	**01:03:01:01** **N/A** **N/A** **N/A**	**N/A** **N/A** **N/A** **01:01:01**	**03:01:01:01** **02:01:01** **03:02:01:01** **N/A**	**03:01:01:01** **06:02:01:01** **06:03:01:01** **N/A**
**DRGATALVLQVLEVPRLM**	**04:02:01** **04:01:01:01** **N/A** **N/A**	**13:01:01:01** **15:01:01:01** **N/A** **N/A**	**01:01:02:01** **N/A** **N/A** **N/A**	**N/A** **N/A** **N/A** **N/A**	**01:03:01:01** **N/A** **N/A** **N/A**	**N/A** **01:01:01** **N/A** **N/A**	**03:02:01:01** **06:02:01:01** **N/A** **N/A**	**06:03:01:01** **N/A** **N/A** **N/A**
**DRGATALVLQVLEVPRLMTQ**	**13:01:01:01** **N/A** **N/A** **N/A**	**16:01:01** **N/A** **N/A** **N/A**	**01:01:02:01** **N/A** **N/A** **N/A**	**N/A** **N/A** **N/A** **N/A**	**N/A** **N/A** **N/A** **N/A**	**02:02:01** **N/A** **N/A** **N/A**	**05:02:01:01** **N/A** **N/A** **N/A**	**06:03:01:01** **N/A** **N/A** **N/A**
**DRGATALVLQVLEVPRLMTQDCLQ**	**04:01:01:01** **03:01:01:01** **N/A** **N/A**	**11:04:01** **N/A** **N/A** **N/A**	**02:02:01:01** **01:01:02:01** **N/A** **N/A**	**N/A** **N/A** **N/A** **N/A**	**01:03:01:01** **N/A** **N/A** **N/A**	**N/A** **N/A** **N/A** **N/A**	**03:01:01:01** **02:01:01** **N/A** **N/A**	**N/A** **06:02:01:01** **N/A** **N/A**
**RGATALVLQVLEVPR**	**04:01:01:01** **N/A** **N/A** **N/A**	**15:01:01:01** **N/A** **N/A** **N/A**	**N/A** **N/A** **N/A** **N/A**	**N/A** **N/A** **N/A** **N/A**	**01:03:01:01** **N/A** **N/A** **N/A**	**01:01:01** **N/A** **N/A** **N/A**	**03:02:01:01** **N/A** **N/A** **N/A**	**06:02:01:01** **N/A** **N/A** **N/A**
**RGATALVLQVLEVPRL**	**04:01:01:01** **N/A** **N/A** **N/A**	**15:01:01:01** **N/A** **N/A** **N/A**	**N/A** **N/A** **N/A** **N/A**	**N/A** **N/A** **N/A** **N/A**	**01:03:01:01** **N/A** **N/A** **N/A**	**01:01:01** **N/A** **N/A** **N/A**	**03:02:01:01** **N/A** **N/A** **N/A**	**06:02:01:01** **N/A** **N/A** **N/A**
**GATALVLQVLEVPR**	**04:02:01** **04:01:01:01** **N/A** **N/A**	**13:01:01:01** **15:01:01:01** **N/A** **N/A**	**01:01:02:01** **N/A** **N/A** **N/A**	**N/A** **N/A** **N/A** **N/A**	**01:03:01:01** **N/A** **N/A** **N/A**	**N/A** **01:01:01** **N/A** **N/A**	**03:02:01:01** **06:02:01:01** **N/A** **N/A**	**06:03:01:01** **N/A** **N/A** **N/A**
**GATALVLQVLEVPRL**	**04:02:01** **03:01:01:01** **04:01:01:01** **N/A**	**13:01:01:01** **N/A** **15:01:01:01** **N/A**	**01:01:02:01** **02:02:01:01** **N/A** **N/A**	**N/A** **N/A** **N/A** **N/A**	**01:03:01:01** **N/A** **N/A** **N/A**	**N/A** **N/A** **01:01:01** **N/A**	**03:01:01:01** **02:01:01** **06:02:01:01** **N/A**	**06:03:01:01** **N/A** **N/A** **N/A**
**ATALVLQVLEVPR**	**04:02:01** **04:01:01:01** **N/A** **N/A**	**13:01:01:01** **15:01:01:01** **N/A** **N/A**	**01:01:02:01** **N/A** **N/A** **N/A**	**N/A** **N/A** **N/A** **N/A**	**01:03:01:01** **N/A** **N/A** **N/A**	**N/A** **01:01:01** **N/A** **N/A**	**03:02:01:01** **06:02:01:01** **N/A** **N/A**	**06:03:01:01** **N/A** **N/A** **N/A**
**ATALVLQVLEVPRL**	**04:01:01:01** **N/A** **N/A** **N/A**	**15:01:01:01** **N/A** **N/A** **N/A**	**N/A** **N/A** **N/A** **N/A**	**N/A** **N/A** **N/A** **N/A**	**01:03:01:01** **N/A** **N/A** **N/A**	**01:01:01** **N/A** **N/A** **N/A**	**03:02:01:01** **N/A** **N/A** **N/A**	**06:02:01:01** **N/A** **N/A** **N/A**
**ALVLQVLEVPRLM**	**01:01:01** **N/A** **N/A** **N/A**	**16:01:01** **N/A** **N/A** **N/A**	**N/A** **N/A** **N/A** **N/A**	**N/A** **N/A** **N/A** **N/A**	**N/A** **N/A** **N/A** **N/A**	**02:02:01** **N/A** **N/A** **N/A**	**05:01:01:01** **N/A** **N/A** **N/A**	**05:02:01:01** **N/A** **N/A** **N/A**
**ALVLQVLEVPRLMTQD**	**03:01:01:01** **N/A** **N/A** **N/A**	**11:04:01** **N/A** **N/A** **N/A**	**01:01:02:01** **N/A** **N/A** **N/A**	**02:02:01:01** **N/A** **N/A** **N/A**	**N/A** **N/A** **N/A** **N/A**	**N/A** **N/A** **N/A** **N/A**	**02:01:01** **N/A** **N/A** **N/A**	**06:02:01:01** **N/A** **N/A** **N/A**
**ALVLQVLEVPRLMTQDCLQ**	**03:01:01:01** **N/A** **N/A** **N/A**	**11:04:01** **N/A** **N/A** **N/A**	**01:01:02:01** **N/A** **N/A** **N/A**	**02:02:01:01** **N/A** **N/A** **N/A**	**N/A** **N/A** **N/A** **N/A**	**N/A** **N/A** **N/A** **N/A**	**02:01:01** **N/A** **N/A** **N/A**	**06:02:01:01** **N/A** **N/A** **N/A**
**LVLQVLEVPRLMTQ**	**04:01:01:01** **03:01:01:01** **N/A** **N/A**	**11:04:01** **N/A** **N/A** **N/A**	**02:02:01:01** **01:01:02:01** **N/A** **N/A**	**N/A** **N/A** **N/A** **N/A**	**01:03:01:01** **N/A** **N/A** **N/A**	**N/A** **N/A** **N/A** **N/A**	**03:01:01:01** **02:01:01** **N/A** **N/A**	**N/A** **06:02:01:01** **N/A** **N/A**
**LVLQVLEVPRLMTQD**	**04:01:01:01** **15:01:01:01** **03:01:01:01** **N/A**	**11:04:01** **N/A** **N/A** **N/A**	**02:02:01:01** **N/A** **01:01:02:01** **N/A**	**N/A** **N/A** **N/A** **N/A**	**01:03:01:01** **N/A** **N/A** **N/A**	**N/A** **01:01:01** **N/A** **N/A**	**03:01:01:01** **06:02:01:01** **02:01:01** **N/A**	**N/A** **N/A** **N/A** **N/A**
**LVLQVLEVPRLMTQDC**	**03:01:01:01** **N/A** **N/A** **N/A**	**11:04:01** **N/A** **N/A** **N/A**	**01:01:02:01** **N/A** **N/A** **N/A**	**02:02:01:01** **N/A** **N/A** **N/A**	**N/A** **N/A** **N/A** **N/A**	**N/A** **N/A** **N/A** **N/A**	**02:01:01** **N/A** **N/A** **N/A**	**06:02:01:01** **N/A** **N/A** **N/A**
**LVLQVLEVPRLMTQDCL**	**03:01:01:01** **N/A** **N/A** **N/A**	**11:04:01** **N/A** **N/A** **N/A**	**01:01:02:01** **N/A** **N/A** **N/A**	**02:02:01:01** **N/A** **N/A** **N/A**	**N/A** **N/A** **N/A** **N/A**	**N/A** **N/A** **N/A** **N/A**	**02:01:01** **N/A** **N/A** **N/A**	**06:02:01:01** **N/A** **N/A** **N/A**
**LVLQVLEVPRLMTQDCLQ**	**11:04:01** **N/A** **N/A** **N/A**	**15:01:01:01** **N/A** **N/A** **N/A**	**02:02:01:01** **N/A** **N/A** **N/A**	**N/A** **N/A** **N/A** **N/A**	**N/A** **N/A** **N/A** **N/A**	**01:01:01** **N/A** **N/A**	**03:01:01:01** **N/A** **N/A** **N/A**	**06:02:01:01** **N/A** **N/A** **N/A**
**VLQVLEVPRLMTQ**	**11:04:01** **03:01:01:01** **N/A** **N/A**	**15:01:01:01** **N/A** **N/A** **N/A**	**02:02:01:01** **01:01:02:01** **N/A** **N/A**	**N/A** **N/A** **N/A** **N/A**	**N/A** **N/A** **N/A** **N/A**	**01:01:01** **N/A** **N/A** **N/A**	**03:01:01:01** **02:01:01** **N/A** **N/A**	**06:02:01:01** **N/A** **N/A** **N/A**
**VLQVLEVPRLMTQD**	**04:01:01:01** **15:01:01:01** **03:01:01:01** **N/A**	**11:04:01** **N/A** **N/A** **N/A**	**02:02:01:01** **N/A** **01:01:02:01** **N/A**	**N/A** **N/A** **N/A** **N/A**	**01:03:01:01** **N/A** **N/A** **N/A**	**N/A** **01:01:01** **N/A** **N/A**	**03:01:01:01** **06:02:01:01** **02:01:01** **N/A**	**N/A** **N/A** **N/A** **N/A**
**VLQVLEVPRLMTQDC**	**03:01:01:01** **N/A** **N/A** **N/A**	**11:04:01** **N/A** **N/A** **N/A**	**01:01:02:01** **N/A** **N/A** **N/A**	**02:02:01:01** **N/A** **N/A** **N/A**	**N/A** **N/A** **N/A** **N/A**	**N/A** **N/A** **N/A** **N/A**	**02:01:01** **N/A** **N/A** **N/A**	**06:02:01:01** **N/A** **N/A** **N/A**
**VLQVLEVPRLMTQDCL**	**03:01:01:01** **N/A** **N/A** **N/A**	**11:04:01** **N/A** **N/A** **N/A**	**01:01:02:01** **N/A** **N/A** **N/A**	**02:02:01:01** **N/A** **N/A** **N/A**	**N/A** **N/A** **N/A** **N/A**	**N/A** **N/A** **N/A** **N/A**	**02:01:01** **N/A** **N/A** **N/A**	**06:02:01:01** **N/A** **N/A** **N/A**
**EVPRLMTQDCLQQSRKVG**	**04:01:01:01** **N/A** **N/A** **N/A**	**15:01:01:01** **N/A** **N/A** **N/A**	**N/A** **N/A** **N/A** **N/A**	**N/A** **N/A** **N/A** **N/A**	**01:03:01:01** **N/A** **N/A** **N/A**	**01:01:01** **N/A** **N/A** **N/A**	**03:02:01:01** **N/A** **N/A** **N/A**	**06:02:01:01** **N/A** **N/A** **N/A**

N/A, Not Available.

### Mutant FVIIa peptides identified on APCs from each donor when incubated with the different FVIIa variants

**Figure 5:**
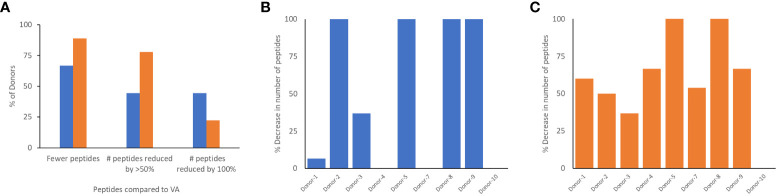
Comparison of peptides identified in the MAPPs assay following incubation of dendritic cells with VA and de-immunized FVIIa proteins. **(A)** The number of peptides from the region of the FVIIa mutation were computed when donor cells were treated with VA, DI-1 (blue), or DI-2 (orange). The graph shows the percent of donors that showed a reduction in the number of peptides when cells were treated with DI-1 or DI-2 compared to VA. We also show the fraction of donors with a 50% and 100% decrease in the number of peptides following treatment with DI-1 or DI-2. In addition, for each of the 11 donors, we depict the per-cent decrease in the number of peptides when treated with DI-1 **(B)** or DI-2 **(C)** compared to VA. Note that VA derived peptides were not identified on some donors thus it is not possible to calculate a decrease in the number of peptides for those donors.

Monocyte-derived Dendritic cells isolated from the same cohort of donors were incubated with the wild-type FVIIa, VA and the two de-immunized variants, DI-1 and DI-2 and subjected to a MAPPs assay (see methods). The FVIIa-derived peptides in the region of the VA mutations (E296V, and M298Q) identified in the MAPPs assay for each of the FVIIa variants are shown in **Tables 2A****–****D**. For each donor we compared the number of peptides identified when the dendritic cells were incubated with VA and each of the two de-immunized variants DI-1 and DI-2. The de-immunized variants were designed to bind with lower affinity to diverse MHC-II variants. For each of the donors in the cohort used we determined: (i) If the mutations introduced in the de-immunized variant resulted in a decrease in binding affinity for the MHC-DRB1 alleles of the donor. (ii) If incubation of the de-immunized variant with the APCs resulted in fewer FVIIa peptides as compared to incubation of VA. The results (**Figure 5A**) show that when incubated with the variants DI-1 and DI-2, 66.7% and 88.9% of donors respectively exhibited fewer FVIIa peptides on the MHC-II proteins. The percent decrease in the number of peptides (compared to VA) for DI-1 and DI-2 are depicted in **Figures 5B, C**, respectively. For the deimmunized variant, D1-1: No peptides were detected when the deimmunized variant was incubated with cells from 4 donors, i.e., a 100% decrease in the number of peptides. There was a 30% decrease in number of peptides following incubation with DI-1 compared to VA for 1 donor. When the experiment was carried out in the remaining 4 donors there was no or minimal (0-10%) decrease in the number of peptides. The deimmunized variant DI-2 too showed a decrease in the number of peptides identified: Only one donor showed no decrease in the number of peptides while the other donors showed a 30-100% decrease in the number of peptides. The mean decrease in the number of peptides presented by DI-1 and DI-2 was 49.3% and 59.3%. The deimmunizing strategy selected mutants that resulted in the largest decrease in the promiscuity scores, i.e., the largest decrease in affinity for the maximum fraction of the population. As illustrated in **Figures 5B, C**, the effect with respect to individual donors can be variable.

### Cluster peptide frequency within donor

**Figure 6:**
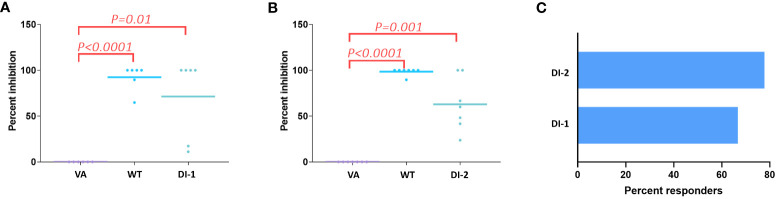
Decrease in cluster peptide frequency for deimmunized FVIIa proteins. Using VA as the positive control we computed the percent inhibition in the cluster frequency (see text) when cells from all 11 donors were treated with the WT FVIIa or DI-1 **(A)** or DI-2 **(B)**. Significant (p-values are depicted on the figure) increases in the percent inhibition of cluster frequencies were observed when cells were treated with WT, DI-1, and DI-2 FVIIa proteins. **(C)** Using cluster frequency as the measure the percent of donors who responded to DI-1 or DI-2 are show. A responder shows a decrease in cluster frequency compared to VA.

Determining the frequency with which peptides of interest occur in a donor offers a biologically relevant parameter. The greater the frequency at which a specific foreign peptide of interest (cluster peptide) is identified the higher the theoretical probability of eliciting an immune response. The cluster-peptide frequencies for each of the FVIIa molecules (wild-type, VA, DI-1, and DI-2) are depicted in **Table 3**. We calculated the percent inhibition in the cluster frequency compared to VA for the wild-type, DI-1, and DI-2 FVIIa proteins (see methods for details). We have shown above, that the wild-type, DI-1, and DI-2 FVIIa proteins exhibit a decrease in T cell proliferation compared to VA (**Figure 1B**). Consistent with this finding, we demonstrate a significant increase in the percent inhibition in the cluster frequency for the wild-type, DI-1, and DI-2 FVIIa molecules compared to VA (**Figures 6A, B**). The percent of donors who showed a decrease in the cluster peptide frequency following deimmunization is depicted in **Figure 6C**.

**Table 3:** Cluster peptide frequency for each variant*.

Variant	Cluster location	Cluster sequence	Frequency within Cohort (%)		Donor 1	Donor 2	Donor 3	Donor 4	Donor 5	Donor 6	Donor 7	Donor 8	Donor 9	Donor 10	Donor 11
**WT**	**291 - 315**	**DRGATALELMVLNVPRLMTQDCLQQ**	**18**	% within Donor	**6**	**0**	**19**	**0**	**0**	**0**	**0**	**0**	**0**	**0**	**0**
**VA**	**289 - 316**	**LLDRGATALVLQVLNVPRLMTQDCLQQS**	**82**		**58**	**38**	**54**	**5**	**7**		**24**	**3**	**6**		**29**
**DI-1**	**283 - 312**	**VSGWGQLLDRGATALVLQVLDVPRLMTQDC**	**45**		**48**	**0**	**48**	**5**	**0**	**0**	**33**	**0**	**0**	**0**	**40**
**DI-2**	**290 - 320**	**LDRGATALVLQVLEVPRLMTQDCLQQSRKVG**	**73**		**30**	**29**	**73**	**2**	**0**	**0**	**14**	**0**	**2**	**13**	**30**

*See text and methods for the definition of cluster peptide frequency.

## Discussion

Both antigen processing and presentation are necessary for eliciting T cell responses. To evaluate these steps of the immune response to therapeutic proteins, conventional methods incubate APCs with the therapeutic protein and/or overlapping peptides derived from the therapeutic protein and then measure T cell responses. The primary drawback of these approaches is that: (i) If over-lapping peptides are used in the assay, many peptides that are found to elicit a T-cell response may not be generated by the proteolytic machinery of the cell (i.e., identification of false positives). (ii) If a protein is used in the assay, it is impossible to determine which of the peptides in the protein elicit the response. A mass spectrometry-based strategy, the so-called MHC-associated peptide proteomics (MAPPs) assay, identifies therapeutic protein derived peptides presented and eluted from the MHC proteins. The assay is finding increasing use in the early non-clinical assessment of therapeutic proteins as it is the only experimental strategy that permits identification of therapeutic-protein derived peptides which are both processed and presented by the immune system. However, the MAPPs assay is both technically demanding and expensive and its value and utility need to be assessed.

To assess the value of any *in vitro* assay to provide results that are predictive of clinical outcomes is difficult. This is because the immunogenicity risk of candidate therapeutic entities using *in vitro* assays are carried out early in drug development. Drug candidates determined to be high-risk are generally not moved forward to clinical studies. Once a candidate drug enters clinical trials it is challenging to obtain samples from patients for the purpose of replicating assays carried out in the non-clinical phase(s). In this study we leveraged an analog of recombinant FVIIa, VA to assess the results from the MAPPs assay. The wild type recombinant FVIIa has been used as a drug for almost 3 decades and there are no reports of immunogenicity for the approved indication. A variant of FVIIa (VA), with an improved safety profile was designed (26) however drug development was discontinued during phase 3 clinical trials because 11% of patients developed anti-drug antibodies (8). In a *post-hoc* study we previously demonstrated that the results of *in vitro* and *ex vivo* assays comparing wild-type FVIIa and VA showed concordance with the clinical outcome (20). We found that 100% of patients with anti-drug antibodies exhibited at least one MHC-II allele that bound with high affinity to VA peptides (compared to 44% of patients with no anti-drug antibodies). T cell–mediated immune responses can be driven by the peptides that bind MHC-II proteins with high affinity. The VA peptide-MHC-II affinity was significantly higher for antidrug antibody positive patients compared to patients which were antidrug antibody negative (no patient with low VA peptide-MHC-II affinity for both HLA-DRB1 alleles developed antidrug antibodies. Taken together, our results indicated T cell mediated development of antidrug antibodies in patients treated with VA. Subsequently, using this information we designed and characterized two de-immunized variants of VA (21) (designated DI-1 and DI-2 in this study).

Compared to the wild-type FVIIa, VA elicited stronger T cell responses (**Figure 1D**) which is consistent with the results of clinical studies (8) and previous *in vitro* findings (20). The DI-1 and DI-2 variants were designed to bind MHCII variants with lower affinity (21) and show T cell responses in a significantly lower number of donors (**Figure 1D**). In these experiments we ensured, (a) that the donor cohort represented 75% of higher frequency DRB1 MHCII variants found in the NA population and (b) that the relative frequencies of the MHCII variants in the donor cohort were comparable to that found in the NA population (**Figure 1C**). Thus, based on the results of clinical studies (8) and *in vitro* assays (**Figure 1**) VA was determined to be a more immunogenic molecule than wild-type FVIIa, DI-1 or DI-2. With this background, wild-type FVIIa, VA, DI-1 and DI-2, which are variants of the same protein but have distinct and well characterized immune responses, were used to evaluate the utility of the MAPPs approach in determining immunogenicity risk for therapeutic proteins.

The donor cohort for the MAPPs assay included MHCII-DRB1 variants found in 75% of the NA population and the more common MHCII-DRB1 variants occur at comparable frequencies in the NA population and in the donor cohort. In general, most peptides identified in the MAPPs assay exhibit high affinity for the MHCII variants identified on the individual donors (**Figure 1D**). We (25) and others (27–29) have shown that peptides identified in MAPPs assays consistently show high affinity for the MHCII variants of the donor and the density plot provides a quality control measure for the MAPPs assay. Similarly, other characteristics of the peptides (e.g., the average, maximum and minimum lengths of the peptides) are also consistent with the biology of MHCII-mediated presentation of exogenous protein-derived peptides.

Numerous peptides, most of which are derived from endogenous proteins expressed and subsequently catabolized by the donor, are identified in a MAPPs assay. From this large dataset we identified the peptides derived from the different FVIIa molecules. We found that for all 4 FVIIa molecules (wild-type, VA, DE-1, and DE-2) 10 clusters of peptides were identified (**Figure 4**). While the relative numbers of peptides differ, all FVIIa molecules present peptides from the same regions of the FVIIa protein. By incubating the different FVIIa molecules with dendritic cells from the same donor cohort, our results suggest comparable protein proteolysis across donors.

From the perspective of immunogenicity risk of therapeutic proteins, foreign peptides that are presented by the MHCII are the ones most likely to drive an immune response (15). As FVIIa is approved for use in hemophilia A patients with inhibitors (23), the patients are not deficient in FVIIa. Consequently, patients who are treated with FVIIa, as well as the donor cohort used in this study, are tolerized to wild-type FVIIa. This postulate is reinforced by the clinical experience (spanning several decades) using recombinant FVIIa wherein there are no reports of immunogenicity for this biologic. However, the introduction of mutations E296V and M298Q into the FVIIa variant (VA) render these peptides foreign. Thus, identification of peptides that include these mutations would be biologically and clinically important. Peptides that include the mutations introduced into VA were detected in Cluster 8 in the heat maps shown in **Figure 4**. The peptides with E296V and M298Q mutations identified in each donor are listed in **Tables 2A****–****D**. This finding provides an important measure of the utility of the MAPPs assay as it provides a mechanistic explanation for the higher incidence of immunogenicity associated with VA (8).

The DI-1 and DI-2 variants of VA offer an alternate approach to evaluating the MAPPs assay. Both variants were specifically designed to decrease the peptide-MHCII binding affinity of the peptides with the E296V and M298Q mutations to diverse MHCII variants. We show that, compared to VA, DI-1 and DI-2 show lower affinity for MHCII DRB1 alleles expressed by the donors in our cohort. Thus, we hypothesize that incubation of DI-1 or DI-2 would result in fewer peptides from Cluster 8 (i.e., those that include the E296V and M298Q mutations introduced into VA). Our results (**Figure 5A**) show that when incubated with the variants DI-1 or DI-2, 66.7% and 88.8% of donors respectively exhibited fewer peptides on the MHC-II proteins (when compared to VA). For DI-1, 45% of donors showed a 100% decrease in the number of peptides identified. For DI-2, 75% showed a 50% reduction in the number of peptides while 25% of donors showed a 100% decrease in the number of peptides (**Figure 5A**). Taken together our data shows that de-immunization of proteins by decreasing the affinity for MHCII alleles results in fewer peptides identified in a MAPPs assay (**Figure 5**) and reduced T-cell proliferation (**Figure 1**).

The cluster frequency is another useful measure that can be obtained from MAPPs data. The frequency that each cluster is presented in the donor cohort is a representation The cluster frequency indicates the percentage of donors that present a specific peptide cluster therefore a high frequency peptide cluster indicates a more promiscuous binding peptide that may indicate a greater risk of immunogenicity. Here, we show that the wild-type FVIIa as well as DI-1 and DI-2 show a significant increase in the per-cent inhibition in cluster frequency compared to VA (**Figure 6**). We determined that 67% and 78% of donors were deemed to respond as expected to DI-1 and DI-2 respectively (i.e., a reduction in the cluster frequency as compared to VA).

The limitations of this study include a relatively small donor cohort of 11 because the MAPPs method is resource intensive and expensive. However, we have endeavored to include a broad and representative set of MHC-II variants (at least with respect to the North American population). Another drawback is that due to the numbers of cells needed and other logistical issues we were unable to carry out the MAPPs assay and T cell proliferation assays on the same donor cohort. As MAPPs assays become more routine, efficient (with respect to the number of cells required) and less expensive more extensive studies to benchmark the results of the MAPPs assay to other *in vitro/ex vivo* methods and clinical outcomes will be possible. We would also like to emphasize that the current study addresses the intrinsic immunogenicity of a therapeutic protein. Many other important variables that are associated with immunogenicity (1) such as impurities, aggregates and leachables from the container and/closure are not studied here.

Here we show that the MAPPs assay used in conjunction with *in-silico* assessments and T cell proliferation assays could provide a useful immunogenicity risk assessment of a candidate protein therapeutic prior to initiation of clinical studies. Additionally, the MAPPs assay allows direct identification of therapeutic protein-derived peptides on HLA variants. These peptides thus represent T cell epitopes which could be relevant for de-immunization programs. We also show that while several scores/parameters can be derived from the MAPPs data, some (e.g., the cluster frequency) show better associations with clinical outcomes.

## Data availability statement

The data presented in the study are deposited in the Harvard Dataverse repository, accession number https://doi.org/10.7910/DVN/7WIZCP.

## Ethics statement

Ethical approval was not required for the studies on humans in accordance with the local legislation and institutional requirements because only commercially available established cell lines were used.

## Author contributions

ZS: Conceptualization, Data curation, Formal Analysis, Funding acquisition, Investigation, Supervision, Writing – original draft, Writing – review & editing. WJ: Conceptualization, Data curation, Formal Analysis, Investigation, Visualization, Writing – original draft, Writing – review & editing. CK: Data curation, Formal Analysis, Visualization, Writing – review & editing. CB: Data curation, Formal Analysis, Investigation, Methodology, Writing – review & editing. EC: Data curation, Formal Analysis, Investigation, Methodology, Writing – review & editing. RR: Data curation, Formal Analysis, Investigation, Methodology, Writing – review & editing.

## References

[1] Sauna ZE Lagasse D Pedras-Vasconcelos J Golding B Rosenberg AS. Evaluating and mitigating the immunogenicity of therapeutic proteins. Trends Biotechnol (2018) 36(10):1068–84. doi: 10.1016/j.tibtech.2018.05.008 29908714

[2] Rosenberg AS Sauna ZE. Immunogenicity assessment during the development of protein therapeutics. J Pharm Pharmacol (2018) 70(5):584–94. doi: 10.1111/jphp.12810 28872677

[3] Shankar G Shores E Wagner C Mire-Sluis A. Scientific and regulatory considerations on the immunogenicity of biologics. Trends Biotechnol (2006) 24(6):274–80. doi: 10.1016/j.tibtech.2006.04.001 16631266

[4] Shankar G Pendley C Stein KE. A risk-based bioanalytical strategy for the assessment of antibody immune responses against biological drugs. Nat Biotechnol (2007) 25(5):555–61. doi: 10.1038/nbt1303 17483842

[5] Gorovits B Wakshull E Pillutla R Xu Y Manning MS Goyal J. Recommendations for the characterization of immunogenicity response to multiple domain biotherapeutics. J Immunol Methods (2014) 408:1–12. doi: 10.1016/j.jim.2014.05.010 24861938

[6] FDA. Guidance for industry: immunogenicity testing of therapeutic protein products - developing and validating assays for anti-drug antibody detection. Silver Spring MD, USA: U.S. Department of Health and Human Services, Food and Drug Administration (2019).

[7] FDA. Guidance for industry: immunogenicity assessment for therapeutic protein products. Silver Spring MD, USA: U.S. Department of Health and Human Services, Food and Drug Administration (2014).

[8] Mahlangu JN Weldingh KN Lentz SR Kaicker S Karim FA Matsushita T. Changes in the amino acid sequence of the recombinant human factor VIIa analog, vatreptacog alfa, are associated with clinical immunogenicity. J Thromb Haemost. (2015) 13(11):1989–98. doi: 10.1111/jth.13141 26362483

[9] Casadevall N Nataf J Viron B Kolta A Kiladjian JJ Martin-Dupont P. Pure red-cell aplasia and antierythropoietin antibodies in patients treated with recombinant erythropoietin. N Engl J Med (2002) 346(7):469–75. doi: 10.1056/NEJMoa011931 11844847

[10] Li J Yang C Xia Y Bertino A Glaspy J Roberts M. Thrombocytopenia caused by the development of antibodies to thrombopoietin. Blood. (2001) 98(12):3241–8. doi: 10.1182/blood.V98.12.3241 11719360

[11] Ridker PM Tardif JC Amarenco P Duggan W Glynn RJ Jukema JW. Lipid-reduction variability and antidrug-antibody formation with bococizumab. N Engl J Med (2017) 376(16):1517–26. doi: 10.1056/NEJMoa1614062 28304227

[12] Lagasse HAD McCormick Q Sauna ZE. Secondary failure: immune responses to approved protein therapeutics. Trends Mol Med (2021) 27(11):1074–83. doi: 10.1016/j.molmed.2021.08.003 34493437

[13] Sauna ZE Richards SM Maillere B Jury EC Rosenberg AS. Editorial: immunogenicity of proteins used as therapeutics. Front Immunol (2020) 11:614856. doi: 10.3389/fimmu.2020.614856 33193460 PMC7658604

[14] Jensen KK Andreatta M Marcatili P Buus S Greenbaum JA Yan Z. Improved methods for predicting peptide binding affinity to MHC class II molecules. Immunology. (2018) 154(3):394–406. doi: 10.1111/imm.12889 29315598 PMC6002223

[15] Jawa V Cousens LP Awwad M Wakshull E Kropshofer H De Groot AS. T-cell dependent immunogenicity of protein therapeutics: Preclinical assessment and mitigation. Clin Immunol (2013) 149(3):534–55. doi: 10.1016/j.clim.2013.09.006 24263283

[16] Roche PA Furuta K. The ins and outs of MHC class II-mediated antigen processing and presentation. Nat Rev Immunol (2015) 15(4):203–16. doi: 10.1038/nri3818 PMC6314495 25720354

[17] Vyas JM van der Veen AG Ploegh HL. The known unknowns of antigen processing and presentation. Nat Rev Immunol (2008) 8(8):607–18. doi: 10.1038/nri2368 PMC2735460 18641646

[18] Karle AC. Applying MAPPs assays to assess drug immunogenicity. Front Immunol (2020) 11:698. doi: 10.3389/fimmu.2020.00698 32373128 PMC7186346

[19] Scharrer I. Recombinant factor VIIa for patients with inhibitors to factor VIII or IX or factor VII deficiency. Haemophilia. (1999) 5(4):253–9. doi: 10.1046/j.1365-2516.1999.00319.x 10469179

[20] Lamberth K Reedtz-Runge SL Simon J Klementyeva K Pandey GS Padkjaer SB. *Post hoc* assessment of the immunogenicity of bioengineered factor VIIa demonstrates the use of preclinical tools. Sci Transl Med (2017) 9(372):eaag1286. doi: 10.1126/scitranslmed.aag1286 28077675

[21] Jankowski W McGill J Lagasse HAD Surov S Bembridge G Bunce C. Mitigation of T-cell dependent immunogenicity by reengineering factor VIIa analogue. Blood Adv (2019) 3(17):2668–78. doi: 10.1182/bloodadvances.2019000338 PMC6737420 31506285

[22] Pandey GS Yanover C Howard TE Sauna ZE. Polymorphisms in the F8 gene and MHC-II variants as risk factors for the development of inhibitory anti-factor VIII antibodies during the treatment of hemophilia a: a computational assessment. PloS Comput Biol (2013) 9(5):e1003066. doi: 10.1371/journal.pcbi.1003066 23696725 PMC3656107

[23] Abshire T Kenet G. Recombinant factor VIIa: review of efficacy, dosing regimens and safety in patients with congenital and acquired factor VIII or IX inhibitors. J Thromb Haemost. (2004) 2(6):899–909. doi: 10.1111/j.1538-7836.2004.00759.x 15140125

[24] McGill JR Yogurtcu ON Verthelyi D Yang H Sauna ZE. SampPick: selection of a cohort of subjects matching a population HLA distribution. Front Immunol (2019) 10:2894. doi: 10.3389/fimmu.2019.02894 31921155 PMC6933600

[25] Jankowski W Park Y McGill J Maraskovsky E Hofmann M Diego VP. Peptides identified on monocyte-derived dendritic cells: a marker for clinical immunogenicity to FVIII products. Blood Adv (2019) 3(9):1429–40. doi: 10.1182/bloodadvances.2018030452 PMC6517663 31053570

[26] Persson E Kjalke M Olsen OH. Rational design of coagulation factor VIIa variants with substantially increased intrinsic activity. Proc Natl Acad Sci U S A. (2001) 98(24):13583–8. doi: 10.1073/pnas.241339498 PMC61084 11698657

[27] Meunier S Hamze M Karle A de Bourayne M Gdoura A Spindeldreher S. Impact of human sequences in variable domains of therapeutic antibodies on the location of CD4 T-cell epitopes. Cell Mol Immunol (2020) 17(6):656–8. doi: 10.1038/s41423-019-0304-3 PMC7264247 31659246

[28] Sekiguchi N Kubo C Takahashi A Muraoka K Takeiri A Ito S. MHC-associated peptide proteomics enabling highly sensitive detection of immunogenic sequences for the development of therapeutic antibodies with low immunogenicity. MAbs. (2018) 10(8):1168–81. doi: 10.1080/19420862.2018.1518888 PMC6284561 30199322

[29] Cassotta A Mikol V Bertrand T Pouzieux S Le Parc J Ferrari P. A single T cell epitope drives the neutralizing anti-drug antibody response to natalizumab in multiple sclerosis patients. Nat Med (2019) 25(9):1402–7. doi: 10.1038/s41591-019-0568-2 PMC6795539 31501610

